# Primary Nonadherence to Antipsychotic Treatment Among Persons with Schizophrenia

**DOI:** 10.1093/schbul/sbac014

**Published:** 2022-03-07

**Authors:** Johannes Lieslehto, Jari Tiihonen, Markku Lähteenvuo, Antti Tanskanen, Heidi Taipale

**Affiliations:** University of Eastern Finland, Department of Forensic Psychiatry, Niuvanniemi Hospital, Kuopio, Finland; University of Eastern Finland, Department of Forensic Psychiatry, Niuvanniemi Hospital, Kuopio, Finland; Karolinska Institutet, Department of Clinical Neuroscience, Stockholm, Sweden; Center for Psychiatry Research, Stockholm City Council, Stockholm, Sweden; University of Eastern Finland, Department of Forensic Psychiatry, Niuvanniemi Hospital, Kuopio, Finland; University of Eastern Finland, Department of Forensic Psychiatry, Niuvanniemi Hospital, Kuopio, Finland; Karolinska Institutet, Department of Clinical Neuroscience, Stockholm, Sweden; University of Eastern Finland, Department of Forensic Psychiatry, Niuvanniemi Hospital, Kuopio, Finland; Karolinska Institutet, Department of Clinical Neuroscience, Stockholm, Sweden; University of Eastern Finland, School of Pharmacy, Kuopio, Finland

**Keywords:** adherence, antipsychotic treatment, schizophrenia

## Abstract

It has remained unclear what factors relate to primary nonadherence to antipsychotic treatment and whether specific agents and routes of administration differ in how patients adhere to them. We collected electronic prescriptions and their dispensings from the Finnish electronic prescription database for 29 956 patients with schizophrenia prescribed antipsychotics via electronic prescription during 2015–2016. We defined primary nonadherence as being prescribed an antipsychotic, which was not dispensed from the pharmacy within one year from prescription. Using logistic regression, we analyzed whether several sociodemographic and clinical factors related to nonadherence. We found that 31.7% (*N* = 9506) of the patients demonstrated primary nonadherence to any of their prescribed antipsychotics. We found that young age (OR = 1.77, 95%CI = 1.59–1.96), concomitant benzodiazepines (OR = 1.47, 95%CI = 1.40–1.55) and mood stabilizers (OR = 1.29, 95%CI = 1.21–1.36), substance abuse (OR = 1.26 95%CI = 1.19–1.35), previous suicide attempt (OR = 1.21, 95%CI = 1.11–1.31), diabetes (OR = 1.15, 95%CI = 1.06–1.25), asthma/COPD (OR = 1.14, 95%CI = 1.04–1.25), and cardiovascular disease (OR = 1.12, 95%CI = 1.05–1.19), were related to primary nonadherence to antipsychotic treatment. Patients using clozapine showed the lowest nonadherence (4.77%, 95%CI = 4.66–4.89), and patients using long-acting injectables were more adherent to treatment (7.27%, 95%CI = 6.85–7.71) when compared to respective oral agents (10.26%, 95%CI = 10.02–10.49). These results suggest that selection between different pharmacological agents and routes of administration while taking into account patients’ concomitant medications (benzodiazepines in particular) and comorbidities play a key role in primary nonadherence to antipsychotic treatment.

## Introduction

Assessing adherence to antipsychotic treatment in clinical care is essential since poor treatment adherence results in poor outcomes in schizophrenia. Specifically, longitudinal studies have found that being without antipsychotic treatment increases the risk of psychotic relapses and is associated with criminality, substance use, poor cognition, suicidal behavior, and overall mortality.^1–6^ Conversely, improved adherence over time relates to improved quality of life in schizophrenia.^7^

It has been estimated that even as few as 40 to 60% of patients with schizophrenia follow their antipsychotic treatment as prescribed.^8–10^ However, these estimates vary vastly depending on the methods used, as one previous systematic review pooling 103 studies (23 796 patients) estimated nonadherence to antipsychotic medication as 25%,^11^ which is only slightly elevated as compared to nonadherence to drugs used in other fields of medicine (about 20%).^8^ Notably, another systematic review found that up to 77% of the previous work on antipsychotic medication adherence is based on subjective and indirect methods (e.g., reports from patients or caregivers)^12^ that potentially overestimate real-life adherence. Specifically, more objective methods (e.g., pharmacy refill records or blood tests) give significantly lower estimates for adherence when compared with the data acquired from subjective sources.^13^ In addition, much of the previous work is primarily based on relatively small^12^ and potentially selected samples, thereby limiting the generalizability of the findings.

Another challenge with the small sample sizes relates to reliably assessing the level of nonadherence for separate antipsychotics since such analysis requires thousands of patients. Although antipsychotics slightly differ in efficacy, the main differences relate to their side-effects.^14^ Further, the most efficacious drugs often induce several unpleasant and potentially severe side-effects such as cardiometabolic side-effects (e.g., clozapine and olanzapine)^14^ or agranulocytosis (clozapine)^15^ that potentially diminish adherence to these drugs. To the best of our knowledge, only one previous large-scale pharmacy refill register study (*N* = 63 214) has assessed adherence to individual antipsychotic drugs.^16^ In their study from 1998 to 1999, of all the explored antipsychotic drugs, patients with schizophrenia had the highest adherence to clozapine, while quetiapine measured the lowest adherence estimate. An update on this question, however, is needed since new antipsychotic drugs that have relatively few side-effects (e.g., aripiprazole) and long-acting injectables (LAIs), which relate to fewer relapses^17^ and lower mortality^18^ when compared with respective oral agents, were not available at the time of the study. Lastly, patients from Valenstein et al., 2004, were military veterans and were mainly males, potentially limiting these findings’ generalizability.

In addition to balancing efficacy and tolerability, a clear understanding of nonadherence to antipsychotic treatment is crucial for informed decision-making in clinical care since medication not taken as prescribed results in suboptimal treatment effectiveness. Here we focused on a subclass of a broad concept of nonadherence, namely primary nonadherence, resulting when a prescription is left undispensed within a period of the initial prescription.^19^ By utilizing large Finnish nationwide databases, we aimed to investigate primary nonadherence to antipsychotic medication in patients with schizophrenia at outpatient care. Besides characterizing the degree and factors relating to primary nonadherence to antipsychotic treatment, we also aimed to assess the degree of primary nonadherence for different routes of administration (i.e., oral or LAI).

## Methods

### Study Base and Data Sources

We included all persons diagnosed with schizophrenia from 1972–2014 in Finland who were alive on January 1, 2015, and aged <65 years. We identified persons with broadly defined schizophrenia from the Hospital Discharge register by the International Classification of Diseases [ICD] version 10 codes F20 and F25 [ICD-10] and ICD-8 and -9 codes 295. Thus, our sample consisted of patients with schizophrenia and schizoaffective disorder.

For the present study, we selected persons in outpatient care who were prescribed antipsychotics via electronic prescription during 2015–2016 (*N* = 29 956) since medications used in hospitals are not recorded in the registers. We collected electronic prescriptions and their dispenses from Kanta, the Finnish electronic prescription database that includes prescriptions used in outpatient care. These prescriptions comprise those prescribed for the first time and renewed ones. The majority of the prescriptions likely represent medications used by the patient before, either in outpatient care or during inpatient hospital stays. Electronic prescriptions became obligatory in 2017, meaning that all prescriptions must be prescribed electronically and saved to the nationwide Kanta database. In case of technical difficulties or emergencies, traditional prescriptions may also be issued, but those are recorded in the Kanta database when dispensed from the pharmacy and, thus, turned into electronic format (no paper versions are allowed anymore). During 2015–2016, electronic prescriptions already covered around 94% of all prescriptions, and mainly prescriptions from small private sector prescriber offices were missing. Hence, all electronic prescriptions are dispensed electronically, and there is no missing data on these dispensings. We also collected the data for the study population on their diagnoses from inpatient and specialized outpatient care registers and Statistics Finland.

### Outcome

In this study, our main outcome measure was primary nonadherence to prescribed antipsychotics, identified by Anatomical Therapeutic Chemical (ATC) classification code N05A (excluding lithium N05AN01). We excluded those antipsychotic prescriptions issued for “as needed” use according to the dose text field of the prescription. For drug-specific analyses, we also categorized antipsychotics according to drug form, either oral or LAI antipsychotics.

We defined primary nonadherence to antipsychotics as being prescribed an antipsychotic, which was not dispensed from the pharmacy within one year. Prescriptions were valid for one year after prescribing, and the follow-up for dispensing in this study covered the years 2015–2017. These electronic prescriptions covered all prescriptions, both for new initiations and renewed prescriptions of drugs used before. The marketing approval of clozapine restricts the one-time prescription and dispensing of clozapine to a maximum of the interval between two monitoring blood samples (due to risk of agranulocytosis), i.e., one month. For comparison purposes, we addressed nonadherence to three other medication groups, namely oral antidiabetics (A10B), antihypertensives (diuretics C03, beta-blockers C07, calcium channel blockers C08, agents acting on the renin-angiotensin system C09), and statins (C10AA). On drug category level, nonadherence means that a person had at least one prescription, which was not dispensed.

### Covariates

Covariates were measured on January 1, 2015, and included age, gender, and time since first schizophrenia diagnosis (≤5, 6–10, 11–20 vs. >20 years). We extracted concomitant medication use from dispensed electronic prescriptions during the study period that covered benzodiazepines and related drugs (N05BA, N05CD, N05CF), antidepressants (N06A), and mood stabilizers (carbamazepine N03AF01, valproic acid N03AG01, lamotrigine N03AX09, lithium N05AN01). We identified comorbid conditions from inpatient (since 1995), and specialized outpatient care (since 1998) registers as cardiovascular disease (ICD-10 I00-I99), diabetes (E10-E14), asthma/COPD (J44-J46), previous suicide attempt (X60-84 and Y10-34), and substance use disorders (F10-F19).

### Statistical Analyses

We used SAS version 9.4 and R version 3.6.1 (https://cran.r-project.org) accompanied with “DMwR”,^20^ “ggplot2” ^21^ packages for statistical analyses and visualizations of the present study. For each specific antipsychotic (categorized into oral and LAI), we calculated the nonadherence proportion by dividing the number of nondispensed prescriptions by the total number of prescriptions. We also calculated a 95% confidence interval (CI) for this proportion. We categorized antipsychotics with <500 prescriptions as “other antipsychotics” in the study population. We used χ ^2^-test to compare dispensed (vs. nondispensed) drugs between agents available in both oral and LAI (e.g., aripiprazole).

Sociodemographic and clinical factors associated with antipsychotic primary nonadherence were assessed with logistic regression and reported as Odds Ratios (ORs) with 95% confidence intervals (CIs). In addition to univariate results, we conducted a multivariate model adjusted for the covariates (effect sizes as adjusted ORs [aORs]), as mentioned earlier. We used the Benjamini-Hochberg False Discovery Rate (FDR) correction^22^ to control false positives due to multiple comparisons.

Lastly, we aimed to test whether the level of primary nonadherence between oral antipsychotics varies as a function of efficacy or tolerability. For this purpose, we used the results from a previous network meta-analysis (402 studies, 53 463 patients with schizophrenia) that compared the previous placebo-controlled and head-to-head randomized controlled trials (RCTs) of different oral antipsychotics.^14^ Briefly, based on the findings of this meta-analysis, we were able to rank different antipsychotics for a given efficacy (e.g., reduction of positive symptoms) or tolerability value (e.g., weight gain). We used *K Nearest Neighbor* to impute missing efficacy or tolerability values for individual antipsychotics (supplementary tables 1 and 2). For efficacy measures, we did not incorporate social functioning, given that most of the explored drugs were missing this value. Next, we ranked efficacy/tolerability estimates and calculated a median of the ranks for each medication, which was also ranked, resulting in lists of overall efficacy and tolerability. Given the cross-sectional nature of our study, the number of users per drug is also potentially driven by efficacy or tolerability. Therefore, as a nonadherence estimate for each oral antipsychotic, we used the ratio of the percentage of nondispensed prescriptions and number of users to account for possible preselection of each medication that is reflected by the number of users per medication. Due to skewed distribution, we used a logarithmically transformed number of users. Finally, we investigated the relationships between the ranked adherence ratio with ranked overall efficacy and tolerability estimates using linear regression. Note, therefore, that we conducted these comparisons at the level of each oral antipsychotic and not at the individual patient’s level. For tolerability, we analyzed the results with and without clozapine because the results of the previous meta-analysis did not incorporate clozapine-specific side-effects such as agranulocytosis or myocarditis that may have a significant effect on tolerability.^15^

## Results

### Sociodemographic and Clinical Characteristics of the Sample

Half of the sample were of male gender (*N* = 15 473), and the average age was 41.8 (table 1). Around half of the patients (i.e., 49.4%, *N* = 14 786) were diagnosed over 20 years ago. About a third of the sample (31.7%, *N* = 9506) were nonadherent to any antipsychotic treatment (i.e., at least one nondispensed prescription). In the sample, 25.1% (*N* = 7505) had cardiovascular disease, 11.0% (*N* = 3294) diabetes, 7.8% (*N* = 2350) asthma or COPD, and 10.7% (*N* = 3215) had attempted suicide previously. Concomitant psychiatric medications were frequent as 46.7% (*N* = 14 002) used benzodiazepines and related drugs, 35.5% (*N* = 10 645) antidepressants, and 24.2% (*N* = 7261) mood stabilizers. In the sample, 22.6% (*N* = 6759) were using antidiabetics, 44.4% (*N* = 13 297) antihypertensives and 25.8% (*N* = 7717) statins, while primary nonadherence to these treatments were 18.3% (*N* = 1239), 20.8% (*N* = 2761), and 13.6% (*N* = 1050), respectively.

**Table 1.** Characteristics of the Study Population (*N* = 29 956)

Variable	
Mean age (SD)	41.8 (13.2)
Age categories, years %(*N*)	
≤25	13.6 (4068)
26–35	16.8 (5040)
36–45	24.0 (7190)
46–55	27.3 (8189)
>55	18.3 (5469)
Male gender %(*N*)	51.7 (15 473)
Time since diagnoses, years %(*N*)	
≤5	10.7 (3203)
6–10	12.8 (3832)
11–20	27.2 (8135)
>20	49.4 (14 786)
Primary nonadherence to any antipsychotic %(*N*)	31.7 (9506)
Comorbid conditions %(*N*)	
Cardiovascular disease %(*N*)	25.1 (7505)
Diabetes %(*N*)	11.0 (3294)
Asthma/ COPD %(*N*)	7.8 (2350)
Substance abuse %(*N*)	21.2 (6358)
Previous suicide attempt %(*N*)	10.7 (3215)
Other medication use %(*N*)	
Benzodiazepine and related drug use	46.7 (14 002)
Antidepressant use	35.5 (10 645)
Mood stabilizer use	24.2 (7261)

### Factors Associated with Primary Nonadherence

Of the sociodemographic factors (table 2), we found that young age (i.e., less than 25 years [aOR = 1.77, 95%CI = 1.59–1.96] and age of less than 35 years [aOR = 1.26, 95%CI = 1.15–1.39]), female gender (aOR = 1.13, 95%CI = 1.08–1.19) and having less than five years since the diagnosis of schizophrenia (aOR = 1.13, 95%CI = 1.08–1.19) related to primary nonadherence. All the explored comorbidities also linked to primary nonadherence: cardiovascular disease (aOR = 1.12, 95%CI = 1.05–1.19), diabetes (aOR = 1.15, 95%CI = 1.06–1.25), asthma/COPD (OR = 1.14, 95%CI = 1.04–1.25), substance abuse (aOR = 1.26 95%CI = 1.19–1.35) and previous suicide attempt (aOR = 1.21, 95%CI = 1.11–1.31). Of the concomitant psychiatric drugs, benzodiazepines (aOR = 1.47, 95%CI = 1.40–1.55) and mood stabilizers (aOR = 1.29, 95%CI = 1.21–1.36) were related to primary nonadherence. All of the above-mentioned relationships remained significant when FDR-corrected. Lastly, we did not find a relation between primary nonadherence and antidepressant use (aOR = 1.02, 95%CI = 0.97–1.08).

**Table 2.** Factors Associated with Primary Nonadherence to Antipsychotics

	Adherent *N* = 20 450	Nonadherent *N* = 9506	Unadjusted OR (95% CI)	Adjusted OR (95% CI)^a^	FDR-corrected *P*-value (Adjusted model)
Age %(*N*)					
≤25	11.3 (2300)	18.6 (1768)	1.90 (1.74–2.07)	1.77 (1.59–1.96)	.0002
26–35	16.4 (3349)	17.8 (1691)	1.25 (1.15–1.35)	1.26 (1.15–1.39)	.0002
36–45	24.9 (5101)	22.0 (2089)	1.01 (0.94–1.09)	1.01 (0.93–1.10)	.7895
46–55	28.4 (5808)	25.1 (2381)	1.01 (0.94–1.09)	1.01 (0.94–1.09)	.7895
>55	19.0 (3892)	16.6 (1577)	reference	reference	
Female gender %(N)	47.6 (9738)	49.9 (4745)	1.10 (1.04–1.15)	1.13 (1.08–1.19)	.0002
Time since diagnoses %(*N*)					
≤5	8.9 (1811)	14.6 (1392)	1.90 (1.75–2.05)	1.40 (1.27–1.53)	0.0002
6–10	12.4 (2533)	13.7 (1299)	1.27 (1.17–1.37)	0.97 (0.89–1.06)	0.5269
11–20	27.3 (5585)	26.8 (2550)	1.13 (1.06–1.20)	0.95 (0.89–1.02)	0.2272
>20	51.5 (10 521)	44.9 (4265)	reference	reference	
Cardiovascular disease %*(N*)	24.4 (4991)	26.5 (2514)	1.11 (1.05–1.18)	1.12 (1.05–1.19)	.0007
Diabetes %*(N*)	10.6 (2175)	11.8 (1119)	1.12 (1.04–1.21)	1.15 (1.06–1.25)	.0013
Asthma/ COPD %(*N*)	7.3 (1489)	9.1 (861)	1.27 (1.16–1.38)	1.14 (1.04–1.25)	.0068
Substance abuse %(*N*)	19.0 (3891)	26.0 (2467)	1.49 (1.41–1.58)	1.26 (1.19–1.35)	.0002
Previous suicide attempt %(*N*)	9.3 (1898)	13.9 (1317)	1.57 (1.46–1.69)	1.21 (1.11–1.31)	.0002
Benzodiazepine use %(*N*)	43.3 (8847)	54.2 (5155)	1.55 (1.48–1.63)	1.47 (1.40–1.55)	.0002
Antidepressant use %(*N*)	34.2 (6987)	38.5 (3658)	1.21 (1.15–1.27)	1.02 (0.97–1.08)	.4944
Mood stabilizer use %(*N*)	22.3 (4559)	28.4 (2702)	1.38 (1.31–1.46)	1.29 (1.21–1.36)	.0002

^a^Adjusted for all covariates listed in this table. COPD, chronic obstructive pulmonary disease.

### The Difference in Primary Nonadherence between Different Antipsychotic Treatments

Table 3 represents the number of users and number of nondispensed prescriptions. There were 29,956 patients with schizophrenia using various antipsychotic medications in the total sample. Of all the explored drugs, we found the highest number of users for oral olanzapine (*N* = 9288) and clozapine (*N* = 8699), and the lowest for sulpiride (*N* = 303) and LAI perphenazine (*N* = 374). Of all antipsychotic prescriptions, 7.39% had not been dispensed. Regarding primary nonadherence to individual antipsychotic treatments, clozapine had a significantly lower number of nondispensed prescriptions (4.77%) than the second-lowest (LAI risperidone, 5.47%): χ ^2^ = 5.29, *P*-value = .02. The highest number of nondispensed prescriptions of all prescriptions were detected for oral haloperidol (17.03%), followed by oral quetiapine (11.39%).

**Table 3.** Primary Nonadherence to Antipsychotic Prescriptions, as a Proportion of Nondispensed Prescriptions of all Prescriptions Issued in 2015–2016

Medication	*N* of Users	*N* of Prescriptions	*N* of Nondispensed Prescriptions	Primary Nonadherence % (95% CI)
Clozapine	8699	126 763	6049	4.77 (4.66–4.89)
Risperidone LAI	1862	5301	290	5.47 (4.89–6.12)
Zuclopenthixol LAI	1049	2578	149	5.78 (4.94–6.75)
Ziprasidone	480	1533	116	7.57 (6.35–9.00)
Perphenazine	2151	5990	455	7.60 (6.95–8.29)
Haloperidol LAI	426	1092	88	8.06 (6.59–9.82)
Chlorprothixene	1693	4461	362	8.11 (7.35–8.95)
Perphenazine LAI	374	973	80	8.22 (6.66–10.12)
Sertindole	392	1289	109	8.46 (7.06–10.10)
Sulpiride	303	872	77	8.83 (7.12–10.90)
Zuclopenthixol	585	1649	149	9.04 (7.75–10.52)
Aripiprazole LAI	649	1535	142	9.25 (7.90–10.80)
Aripiprazole	4541	12 893	1254	9.73 (9.23–10.25)
Other antipsychotic	494	1198	117	9.77 (8.21–11.58)
Levomepromazine	2759	7305	752	10.29 (9.62–11.01)
Olanzapine	9288	30 311	3127	10.32 (9.98–10.66)
Risperidone	4036	11 031	1143	10.36 (9.81–10.94)
Olanzapine LAI	913	2681	280	10.44 (9.34–11.66)
Paliperidone LAI	1110	3268	347	10.62 (9.61–11.72)
Quetiapine	7293	26 510	3019	11.39 (11.01–11.78)
Haloperidol	1164	3217	548	17.03 (15.77–18.37)
Total	29 956	252 450	18 653	7.39 (7.29–7.49)

As a group, oral antipsychotics measured a higher number of nondispensed prescriptions (10.26%, 95% CI = 10.02–10.49) compared to their LAI counterparts (7.27%, 95%CI = 6.85–7.71), χ ^2^ = 118.08, *P*-value < .0001. When we explored individual agents, lower number of nondispensed prescriptions for LAIs (vs. orals) were observed for haloperidol (χ ^2^ = 51.50, *P*-value < .0001), risperidone (χ ^2^ = 106.40, *P*-value < .0001) and zuclopenthixol (χ ^2^ = 15.78, *P*-value < .0001). No such differences were observed for aripiprazole (χ ^2^ = 0.30, *P*-value = .58), olanzapine (χ ^2^ = 0.04, *P*-value = .85) or perphenazine (χ ^2^ = 0.38, *P*-value = .54).

**Fig. 1.**
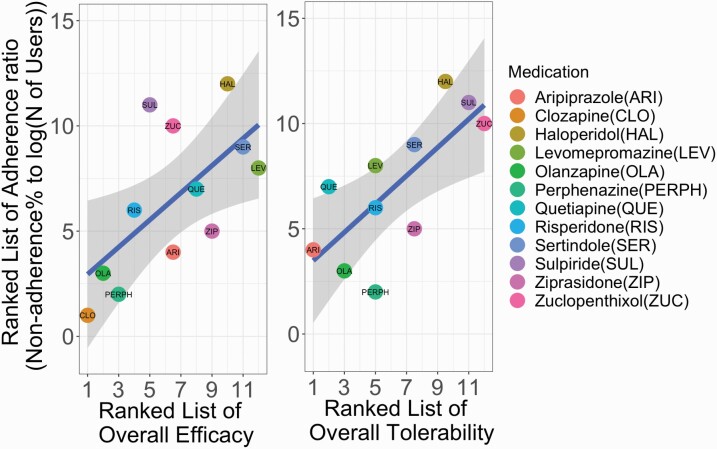
Relationships between the ratio between the percentage of nondispensed prescriptions and the number of users with overall efficacy (left) and overall tolerability (right). Efficacy and tolerability estimates were acquired from Huhn et al. 2019. Analysis of the relationship with tolerability with clozapine included is provided in supplementary figure 1. The blue regression lines are plotted with grey shaded 95% CIs.

Relationships between adherence ratio (i.e., the ratio of the percentage of nondispensed prescriptions and number of users for each antipsychotic medication) for antipsychotics with efficacy and tolerability are shown in figure 1. We found that both efficacy (R^2^ = 0.42, FDR-corrected *P*-value = .024) and tolerability (R^2^ = 0.53, FDR-corrected *P*-value = .021) related to adherence ratio across the explored antipsychotic treatments. These relationships indicate that antipsychotics with a low number of side effects and high efficacy are highly likely to be dispensed when prescribed and used by many patients. The findings regarding tolerability did not remain statistically significant when incorporating clozapine in the analyses (R^2^ = 0.24, *P*-value = .10), as shown in supplementary figure 1.

## Discussion

The present study used nationwide register-based data to assess primary nonadherence to antipsychotic treatment in patients with schizophrenia. Our sample included only patients with schizophrenia that were in outpatient care. Although antipsychotic treatment is efficacious in treating psychotic symptoms,^14^ prevents future relapses^17^ and relates to lower overall mortality in patients with schizophrenia compared with no antipsychotic treatment,^6,23,24^ about a third of the patients, mainly in the chronic stage, demonstrated primary nonadherence to these medications (i.e., at least one unfilled prescription for antipsychotic treatment within the one-year follow-up). The true nonadherence is probably higher than that, as collecting antipsychotic drugs from a pharmacy does not necessarily equate to taking them as prescribed. However, primary nonadherence refers to situations where with high certainty patient has not been using the medication as it was not even dispensed from the pharmacy, and most previous adherence studies have not included this aspect at all. Our finding is in line with a previous American pharmacy refill study reporting that 40% of the patients in outpatient care demonstrate poor adherence. Note, however, that the previously mentioned study used a different method to assess nonadherence (i.e., defined as medication possession ratio < 0.8).^16^ Altogether, these estimates are significantly higher than nonadherence assessed for drugs used in other fields of medicine (about 20%).^8^ Further, schizophrenia patients in our sample demonstrated lower primary nonadherence (14–21%) to somatic medications compared to antipsychotic medications.

There were several relationships between sociodemographic variables with primary nonadherence to antipsychotics. First, we found that female (vs. male) gender was related to higher primary nonadherence, which is a relatively unexpected finding since the previous literature has also reported the opposite relationships to adherence.^11^ Note, however, that some other studies report no relationship between gender and adherence to antipsychotic treatment.^9,16^ The discrepancies mentioned above may relate to differences in the definition of adherence across the previous studies, as there might be gender differences in both primary and secondary nonadherence. In line with the previous work,^2,11^ young age and recently diagnosed schizophrenia were related to primary nonadherence to antipsychotic treatment. The relationship between young age and poor adherence is alarming as patients with early-onset schizophrenia often have poor disorder outcomes (e.g., poor global outcome, frequent relapses, and rehospitalizations) throughout the disorder^25^ and have an increased risk of using illicit substances.^26^

There were also several clinical factors relating to primary nonadherence. The present study found that one in five patients with schizophrenia had a diagnosed substance abuse comorbidity, also related to primary nonadherence to antipsychotic treatment. The relationship between substance abuse and nonadherence is a well-reported finding^9,11,27^ and might stem from a lack of insight and need for antipsychotic treatment while actively using substances. It has been suggested that patients with schizophrenia with comorbid substance abuse aim to self-alleviate dysphoria with illicit substances.^28^ We also found that previous suicide attempts related to poor adherence, which is in line with other observational studies.^4,5,29,30^ This finding is noteworthy because, although the risk has decreased in recent years, patients with schizophrenia still have a high risk of suicide compared to the general population.^31^ It has been discussed in the literature that the experienced side-effects, especially extrapyramidal side-effects, might partly explain this relationship.^32^ Lastly, we found that several somatic comorbidities (i.e., cardiovascular, asthma/COPD, and diabetes) were frequent in patients with primary nonadherence to antipsychotics, potentially reflecting the accumulation of poor lifestyle choices (e.g., smoking, obesity) in this group. Interestingly, prior evidence shows that current antipsychotic use in schizophrenia relates to decreased risk of discontinuation cardiovascular medication.^33^ Combined with our findings, these results indicate that nonadherence to antipsychotics might reflect poor somatic health and contribute to well-reported excessive mortality in schizophrenia.^31^

To our knowledge, this is the first large-scale study to explore the concomitant use of psychotropic medications and primary nonadherence to antipsychotics. We found that about half of the patients with schizophrenia used benzodiazepines. Alarmingly, nonadherence to antipsychotic treatment was related to benzodiazepine use indicating selective usage of psychotropic drugs among patients with schizophrenia with primary nonadherence to antipsychotics. While all the major clinical guidelines recommend only short-term use for acute distress, insomnia, and agitation, previous work has shown that the usage of benzodiazepines often lasts longer than recommended.^34^ Long-term benzodiazepine use is problematic in schizophrenia as there is evidence showing that benzodiazepine use relates to higher mortality in schizophrenia.^6^ Lastly, we found that nonadherence to antipsychotics is associated with concurrent mood stabilizer use, which possibly relates to an increment in medication burden and dosing frequency via the initiation of a new agent. Previous work has shown that increases in dosing frequency relate to a decrease in adherence in patients with schizophrenia.^35^

Alike the previous work,^16,36^ patients with schizophrenia exhibited the highest adherence to clozapine, which is somewhat surprising since clozapine also has the most severe side-effects.^15^ However, given the widely reported results from RCTs^14^ and observational studies^17^ of the superiority of clozapine in efficacy compared to any other oral antipsychotic treatment, our results may reflect the actual effect of the treatment experienced by the patients. On the other hand, due to agranulocytosis risk, clozapine patients are monitored using regular blood tests, implying that our finding may also partially stem from close and regular contact with healthcare. Close contact may enhance conveying the drug-related experiences to healthcare workers, which is important as one of the main complaints is that patients feel that side-effects are not discussed openly.^32^ In fact, a working alliance is the most notable and consistent predictor of adherence to medication recommendations.^36^ Lastly, due to the requirements for strict blood monitoring routines, our result may also partially indicate selection bias as it is also possible that only patients with high compliance start and continue clozapine treatment.

We found that in addition to the administration route, antipsychotic treatment’s efficacy and tolerability appear to be critical factors affecting patients’ adherence to antipsychotic treatment. First, we found that LAIs showed significantly higher adherence estimates than respective oral agents, potentially resulting from sparser administration than oral treatment. Our finding adds to previous work showing that, compared to oral treatment, the usage of LAIs relates to lower mortality and rehospitalization risk in schizophrenia,^16,17^ thereby supporting the usage of LAIs in clinical practice. Factors relating to variations in primary adherence to oral antipsychotics have previously been inconclusive as some studies have found a relation between side-effects and primary nonadherence,^37,38,39^ but there are also studies showing no relationship.^9,40^ Also, the role of poor effectiveness of the treatment on nonadherence has been discussed in the literature.^41^ Previous studies, however, have been conducted in relatively small samples focusing on a few pharmacological agents. Here we found that oral antipsychotics that have been shown to have both relatively few side effects and high efficacy (results from a previous network meta-analysis of the RCTs on antipsychotics)^14^ were dispensed with high probability and used by many patients. Contrary, drugs with a high number of side effects and low efficacy measured low adherence estimates and were used by a small number of patients.

A major strength of our study was a large, nationwide sample of patients with schizophrenia, thereby providing high generalizability of the results to other samples in countries akin to Finland with the state-funded health care system. Secondly, we used electronic records to objectively identify whether or not the prescribed medication was dispensed. All antipsychotic medications are only available with a prescription, and hence, are recorded in the national register. There were also several weaknesses. Here we focused only on primary nonadherence assessed using pharmacy data, which, albeit objective, does not fully capture the degree of nonadherence as some patients may not use their medication as prescribed. Future work should use complementary assessments (e.g., blood tests and pharmacy data) to characterize the level of nonadherence to tackle potential underestimation. Lastly, our work cover only two years’ prescriptions, which might limit the generalization of our study as the adherence to antipsychotic treatment may vary over time.^36^

In conclusion, our study has shown that about a third of patients with schizophrenia demonstrate primary nonadherence to antipsychotic medication. Selection between different pharmacological agents and their routes of administration while taking into account patients’ concomitant medications (benzodiazepines in particular) and comorbidities play a key role in primary nonadherence to antipsychotic treatment.

## Supplementary Material

sbac014_suppl_supplementary_Figure_1

sbac014_suppl_Supplementary_Table_1

sbac014_suppl_Supplementary_Table_2
