# Supplementary figures and images for: Primary Nonadherence to Antipsychotic Treatment Among Persons with Schizophrenia
Source: Schizophr Bull. 2022 Mar 7;48(3):655–63. doi: 10.1093/schbul/sbac014 (PMC9077427; doi:10.1093/schbul/sbac014)

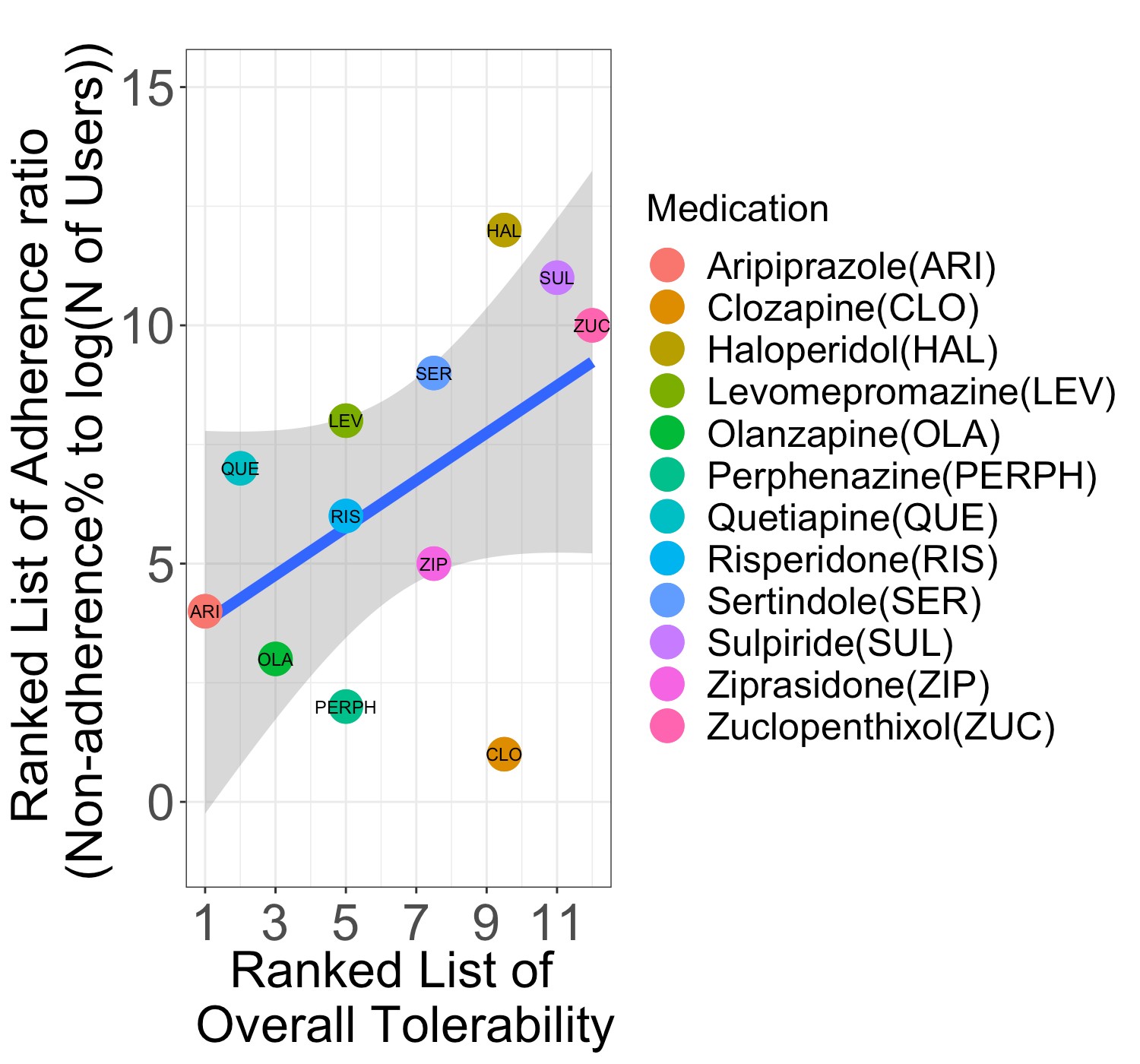

Supplement: sbac014_suppl_supplementary_Figure_1 [file sbac014_suppl_supplementary_figure_1.jpeg]
