# Supplementary material for: Primary Nonadherence to Antipsychotic Treatment Among Persons with Schizophrenia
Source: Schizophr Bull. 2022 Mar 7;48(3):655–63. doi: 10.1093/schbul/sbac014 (PMC9077427; doi:10.1093/schbul/sbac014)
Supplement: sbac014_suppl_Supplementary_Table_1 [file sbac014_suppl_supplementary_table_1.docx]

**Supplementary Table 1.** Ranked list of efficacy estimates based on Huhn et al., 2019. Imputed values in red.

| Medication | Overall Change in Symptoms | Positive Symptoms | Negative Symptoms | Depressive Symptoms | All-Cause Discontinuation | Ranked list |
| --- | --- | --- | --- | --- | --- | --- |
| Clozapine | 1 | 1 | 1 | 2 | 2 | 1 |
| Olanzapine | 2.5 | 3 | 2 | 4 | 1 | 2 |
| Risperidone | 4 | 2 | 4.5 | 7 | 6.5 | 4 |
| Perphenazine | 2.5 | 6 | 3 | 8 | 3 | 3 |
| Zuclopenthixol | 5 | 7.5 | 11 | 11 | 6.5 | 6.5 |
| Haloperidol | 7 | 4 | 10 | 10 | 9.5 | 10 |
| Sulpiride | 6 | 5 | 6 | 1 | 11 | 5 |
| Quetiapine | 8 | 9.5 | 9 | 6 | 8 | 8 |
| Aripiprazole | 9.5 | 11 | 7.5 | 3 | 4 | 6.5 |
| Ziprasidone | 9.5 | 7.5 | 7.5 | 9 | 9.5 | 9 |
| Sertindole | 11 | 9.5 | 4.5 | 12 | 12 | 11 |
| Levomepromazine | 12 | 12 | 12 | 5 | 5 | 12 |
