# Supplementary material for: Primary Nonadherence to Antipsychotic Treatment Among Persons with Schizophrenia
Source: Schizophr Bull. 2022 Mar 7;48(3):655–63. doi: 10.1093/schbul/sbac014 (PMC9077427; doi:10.1093/schbul/sbac014)
Supplement: sbac014_suppl_Supplementary_Table_2 [file sbac014_suppl_supplementary_table_2.docx]

**Supplementary Table 2.** Ranked list of side-effects based on Huhn et al., 2019. Imputed values in red.

| Medication | Weight Gain | Use of Antiparkinson Medication | Akathisia | Prolactine Elevation | QTc Prolongation | Sedation | Anticholinergic Side-Effects | Ranked list |
| --- | --- | --- | --- | --- | --- | --- | --- | --- |
| Clozapine | 8 | 1 | 1 | 1 | 8 | 9 | 10 | 9.5 |
| Olanzapine | 12 | 3 | 3 | 5 | 4 | 7 | 9 | 3 |
| Risperidone | 6 | 8 | 8 | 12 | 5 | 6 | 4 | 5 |
| Perphenazine | 5 | 10 | 10 | 10 | 6 | 1 | 5 | 5 |
| Zuclopenthixol | 3 | 11 | 12 | 8 | 7 | 12 | 11 | 12 |
| Haloperidol | 4 | 12 | 9 | 11 | 2 | 5 | 8 | 9.5 |
| Sulpiride | 10 | 9 | 11 | 9 | 9 | 11 | 1 | 11 |
| Quetiapine | 9 | 4 | 4 | 3 | 3 | 10 | 12 | 2 |
| Aripiprazole | 2 | 5 | 6 | 2 | 1 | 4 | 3 | 1 |
| Ziprasidone | 1 | 7 | 7 | 4 | 11 | 8 | 6 | 7.5 |
| Sertindole | 11 | 2 | 2 | 7 | 12 | 3 | 7 | 7.5 |
| Levomepromazine | 7 | 6 | 5 | 6 | 10 | 2 | 2 | 5 |
